# “Z”-Shaped Rotational Au/Pt Micro-Nanorobot

**DOI:** 10.3390/mi8060183

**Published:** 2017-06-08

**Authors:** Kai Chen, Chenyi Gu, Zhan Yang, Masahiro Nakajima, Tao Chen, Toshio Fukuda

**Affiliations:** 1 Provincial Jiangsu Key Laboratory for Advanced Robotics, Soochow University, Suzhou 215123, China; kchen@stu.suda.edu.cn (K.C.); 20164229001@stu.suda.edu.cn (C.G.); 2 Collaborative Innovation Center of Suzhou Nano Science and Technology, Soochow University, Suzhou 215123, China; 3 Department of Micro-Nano Systems Engineering, Nagoya University, Nagoya 464-8603, Japan; nakajima@mein.nagoya-u.ac.jp (M.N.); fukuda@mein.nagoya-u.ac.jp (T.F.); 4 Intelligent Robotics Institute, School of Mechatronic Engineering, Beijing Institute of Technology, Beijing 100081, China

**Keywords:** nanorobot, self-actuation, drug delivery system

## Abstract

Drug delivery, minimally-invasive surgery, and a hospital-in-the-body are highly desirable for meeting the rapidly growing needs of nanorobot. This paper reports a Z-shaped gold/platinum (Au/Pt) hybrid nanorobot which realizes the self-rotational movement without an external force field. The Z-shaped Au/Pt hybrid nanorobot was fabricated by focused ion beam (FIB) and plasma sputtering. The purity of the nanorobot was tested by energy dispersive X-ray analysis (EDS). The weight percentage of Pt and Au at the tip were 94.28% and 5.72%, respectively. The weight percentage of Pt and Au at the bottom were 17.39% and 82.75%, respectively. The size of the nanorobot was 2.58 × 10^−16^ m^2^ and the mass of the nanorobot was 8.768 × 10^−8^ kg. The driving force of the nanorobot was 9.76 × 10^−14^ N at the 6.9% concentration of hydrogen peroxide solution. The rotation speed was 13 rpm, 14 rpm, and 19 rpm at 5.6%, 6.2%, and 7.8% concentrations, respectively.

## 1. Introduction

The rapidly increasing demands in biomedical health-care and the environment [1,2] are driving the development of nanorobots. Nanorobots promise to reduce the invasiveness of a variety of medical procedures, lower the risk of complications, and result in shorter recovery times. Nanorobot in situ rotation is required for further application of micro-fluid mixing and biological cell transfer. Synthetic nanorobots convert chemical or other forms of energy into mechanical movement, such as electrochemical driving, light driving (near-infrared), ultrasound driving, magnetic driving, etc.

To date most studies of nanorobots have involved chemical propulsion. Hydrogen peroxide decomposition at the surface of catalytic nanorobots generates particles. The extrusion and collision of these particles in a narrow space form the driving force. For example, Schmidt and coworkers made use of rolled-up nanotechnology [3] to fabricate nanotubes consisting of platinum (Pt) and other materials [4]. Hydrogen peroxide catalytically decomposed into oxygen and water while in contact with the Pt. The bubbles aggregated by the oxygen molecules ejected from one end of the nanotube. The nanotube moved at approximately 23 μm/s in 10 times diluted samples of blood at 37 °C, i.e., physiological temperatures [5].

Wang et al. firstly proposed that a Janus particle zoomed around in pure water [6]. Aluminum reduced the water to hydrogen. The detachment of hydrogen bubbles propelled the Janus particle at a very high speed of 3 mm·s^−1^. The reaction process was hindered by a rapidly formed oxide passivation layer on the Al surface. To solve the problem, the Janus particle was manufactured by an aluminum-gallium alloy. The gallium prevented the aluminum from forming the aluminum hydroxide film. However, aluminum, as a reactant, was not sustainable.

The also group utilized strong acid as fuel to drive nanotubes containing zinc [7], but the zinc would be consumed eventually.

Except for self-propelled nanorobots mentioned above, other forms of energy were used. He et al. used near-infrared (NIR) light to drive the nanorobot which was fabricated by a layer-by-layer (LbL) technique [8,9]. The outer and inner material of the nanotube included a polycarbonate membrane and gold nanoshells (AuNSs), respectively. Upon NIR irradiation, the AuNSs of the tubes converts the light energy to thermal energy. The generated thermal energy caused rapid evaporation of water within the tubes which caused an explosion of the tubes. Ultrasound was first used by Hoyos, Mallouk, and coworkers to power swimmers. Resonating ultrasound was converted into directional motion and spin. Wang et al. [10] described a microparticle in a sound field subjected to an acoustic ultrasound, which drove it.

A magnetic field was also used as energy to drive a nanorobot. The nanorobot fabricated with magnetic material was magnetized and driven by the mobile magnetic field. Nelson et al. designed a multi-link nanowire-based nanoswimmer which, for the first time, demonstrated the planar undulations induced by a planar-oscillating magnetic field [11]. Those chains were comprised of an elastic eukaryote-like polypyrrole tail and rigid magnetic nickel links which were connected by flexible polymer bilayer hinges. The flexible tail-like prokaryote flagella fluctuated with the magnetic field of swing, which propelled it.

Bubble propulsion such as microtubes, janus microspheres realized high power output and high speed. However, the production of bubbles is not necessary desirable. Bubbles ejection often leads to irregular trajectories. It is easy to control the speed and directionality of the nanorobot by extra energy field. However, nanorobots move not autonomously without extra equipment.

Commonly, the chemical propulsion included three mechanisms which are self-electrophoresis, chemical reaction, and catalytic reaction. The reacting area and the concentration of the solution were two key factors to the chemical reaction. A Janus microsphere realized high speed, however, the reactant was not sustainable. The catalytic reaction generated bubbles which drove the motion of the nanorobot, however, the driving force of it was non-linear because the generation of the bubble was not homogeneous. It was difficult to control it in planned trajectories. All of them were subjected by the Brownian force which resulted in irregular motion. This article describes a Z-shaped self-rotational nanorobot. The actuation mechanism of the Z-shaped nanorobot was self-electrophoresis [12]. The two reverse forces at both ends and the force arm between them form a couple which drives its in situ rotation. The in situ rotation is regular and stable.

In this paper we designed a gold/platinum nanorobot to construct a chemical cell which catalyzed hydrogen peroxide to actuate the nanorobot. Based on the Au/Pt nanorod, the Z-shaped nanorobot was designed. The two reverse forces at both ends and the force arm between them form a couple which drives its in situ rotation. In Section 2, the mechanism and fabrication of the Z-shaped nanorobot was illustrated. Section 3 shows the manipulation and results of the Z-shaped nanorobot, which mainly demonstrate the release process and the analysis of the rotary motion.

## 2. Mechanism and Fabrication

### 2.1. Mechanism

The Pt/Au hybrid nanorobot was firstly designed by Mallouk et al. at Pennsylvania State University [13]. The nanorobot showed autonomous non-Brownian movement toward the platinum end in a hydrogen peroxide solution. Based on the nanorod, the Z-shaped nanorobot was designed to realize the nanorobot in situ rotation for further application of micro-fluid mixing and biological cell transfer. The two reverse forces at both ends and the force arm between them form a couple which drive its in-situ rotation. Motion of the Z-shaped nanorobot was regular and stable. It was faster than linear motion. According to the experimental phenomenon, various mechanisms had been proposed for the self-propulsion of the bimetallic catalytic nanorobots like the differential pressure, the interfacial tension [14,15], the diffusion-phoresis, etc. Through researching, self-electrophoresis is recognized as the most reasonable mechanism. 

The mechanism was oxidation reduction of the hydrogen peroxide. The reduction reaction on the gold segment and the oxidation reaction on the platinum segment caused electron flux. The electrons migrated from platinum to gold and generated an electric field [16]. Electrochemical reactions taking place on the Pt/Au hybrid nanorobot are shown in Equations (1)–(3):

(1) $$\mathrm{Overall}:2H_{2}O_{2}=2H_{2}O+O_{2},$$

(2) $$\mathrm{Anode}\left(\mathrm{Pt}\right):H_{2}O_{2}=O_{2}+2H^{+}+2e^{-},$$

(3) $$\mathrm{Cathode}\left(\mathrm{Au}\right):H_{2}O_{2}+2H^{+}+2e^{-}=2H_{2}O.$$

**Figure 1:**
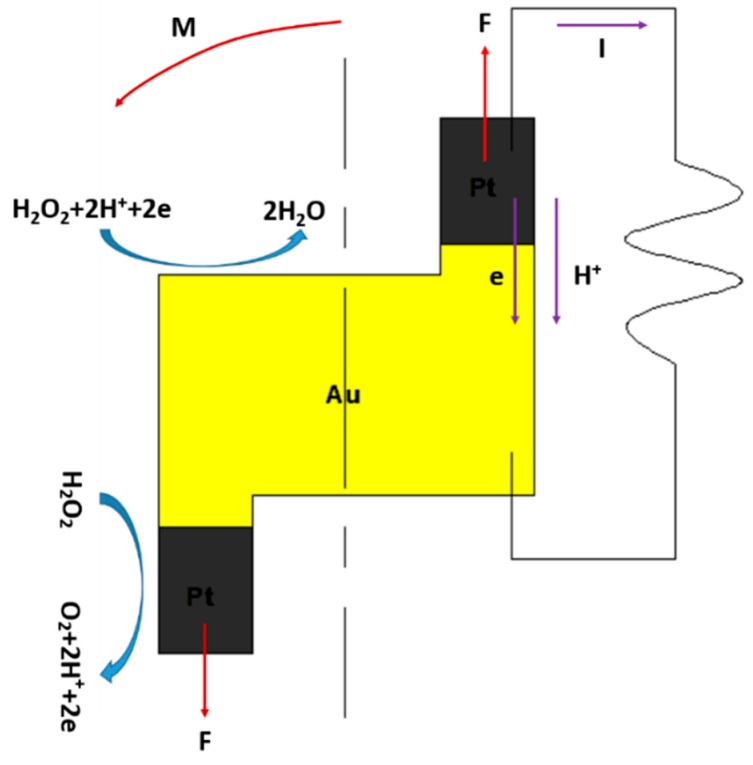
The mechanism of self-electrophoresis of Pt/Au hybrid nanorobot.

The decomposition reaction and combination reaction occurred on the Pt segment and Au segment, respectively. The Pt and Au were not reacting with hydrogen peroxide. Figure 1 shows that the driving force was caused in two ways. One was a molecular concentration gradient. The number of particles on the Au side were twice as much than on the Pt side because of electron immigration, which generated a concentration gradient. The other was the reverse thrust of the ion flux caused by electron immigration from Pt to Au. Two opposite forces on different sides of the moment arm formed a couple which drove its rotation.

### 2.2. Experimental Instrument of Fabrication

The main material was commercial platinum which was used to fabricate the Z-shaped Pt/Au hybrid nanorobot by a focused ion beam (FIB) instrument. The gold layer was coated on the platinum via a sputtering machine, then FIB was used to etch off the superfluous gold layer until the platinum appeared.

The FIB instrument (SMI 2050, Tokyo, Japan) was used. The focused ion beam generated a gallium (Ga^+^) primary ion beam hitting the sample surface and sputtering a small volume of material. The material left the surface with either secondary ions or neutral atoms. The primary beam also produced secondary electrons. The primary beam raster was putted on the sample surface to collect the signal from the sputtered ions or secondary electrons to form an image. The etching operation was that high-energy Ga^+^ struck the sample and they sputtered atoms from the surface.

### 2.3. Process and Result of Fabrication

**Figure 2:**
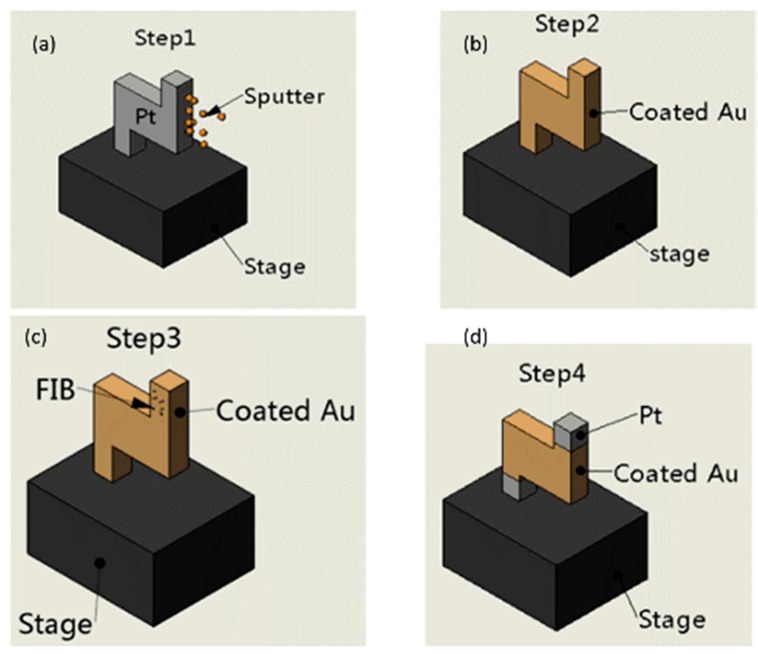
The mechanism of sputtering deposition. (**a**) Gold was coated on the nanorobot via a sputtering machine. (**b**) The nanorobot was fully coated with gold. (**c**) The nanorobot was etched by the FIB. (**d**) The structure of the Z-shaped nanorobot.

The Z-shaped Pt/Au hybrid nanorobot was fabricated by a method combining focused ion beam (FIB) and gold sputtering. The fabrication process is demonstrated in Figure 2. Firstly, the Z-shaped Pt segment was etched by FIB and gold was coated on the Pt segment via a sputtering machine as shown in Figure 2a,b; the Z-shaped segment was fully coated with gold as Figure 2b shows, and then using FIB to etch off the gold layers of the Z-shaped segments, as Figure 2d shows.

**Figure 3:**
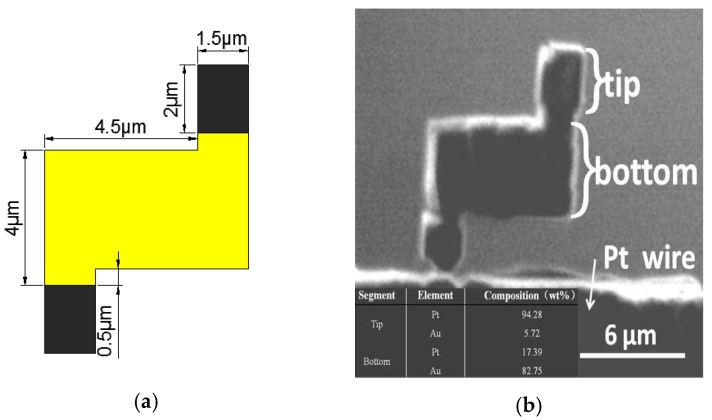
(**a**) The dimension of the Z-shaped nanorobot. (**b**) SEM image of the Z-shaped Au/Pt hybrid nanorobot.

The Figure 3 shows the dimensions and a SEM image of the nanorobot. The Pt/Au hybrid nanorobot was cut off mechanically by the glass capillary. In order to realize this kind of mechanical cut, a narrow neck was etched out by FIB as shown in Figure 3b. As mentioned in Section 2.1, the propulsion for the Pt/Au hybrid nanorobot was self-electrophoresis caused by the electrochemical reaction. Thus, the purity of the gold thin layer was a critical point in this fabrication method. In order to validate the fabrication method, the energy-dispersive X-ray analysis (EDS) was carried out to check the purity of Pt/Au hybrid nanorobot.

### 2.4. Experimental Instrument and Result of Validation

Energy-dispersive X-ray analysis, also known as EDS, EDX, or EDAX, is a technique used to identify the elemental composition of a sample or small area of interest on the sample. During EDS, a sample was exposed to an electron beam inside a scanning electron microscope (SEM). These electrons collide with the electrons within the sample, causing some of them to be knocked out of their orbits. The vacated positions are filled by higher energy electrons which emit X-rays in the process. By analyzing the emitted X-rays, the elemental composition of the sample was determined.

From the above results, it was obvious to see that, at the tip segment, the majority material was Au, which was coated on the surface, and at the tip segment the Au composition was not pure because of the penetration of the electron beam. On the other hand, at the bottom segment, the majority material was Pt where the coated Au layer had been etched away by FIB, and there was some Au contamination remaining, accounting for about 5.72%. Based on the EDS analysis result, the new fabrication method combined with FIB and gold sputtering was validated because the dominant material on two segments was Pt and Au, respectively. Thus, the fabrication also ensured the purity of the Z-shaped Au/Pt hybrid nanorobot.

## 3. Experiment Manipulation and Results

### 3.1. Method and Experimental Instrument of Manipulation

**Figure 4:**
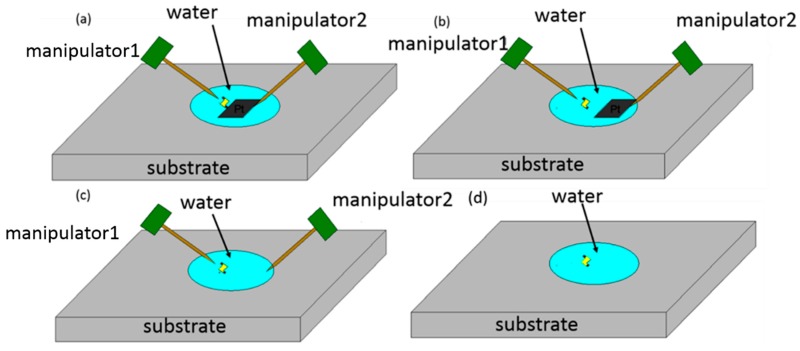
Schematic of manipulation method. (**a**) Cutting off the Z-shaped Pt/Au hybrid nanorobot from the Pt probe. (**b**) Removing the Pt probe stage. (**c**) Releasing the nanorobot into water with a chopsticks-like manipulator [16,17,18,19,20]. (**d**) Injection of hydrogen peroxide solution.

**Figure 5:**
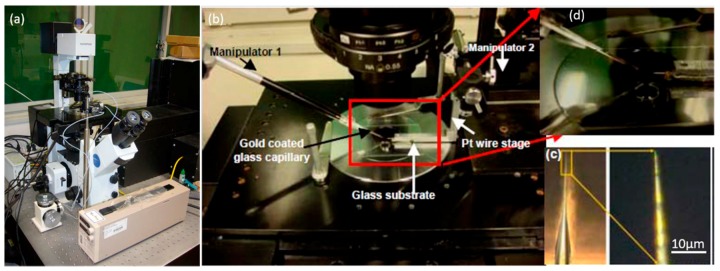
Laboratory equipment. (**a**) Inverted optical microscope (IX71, Olympus). (**b**) The overall view of the setup containing two manipulators, glass capillary, Pt stage, etc. (**c**) Gold-coated glass capillary with 1 µm shaped tip. (**d**) Inset picture of glass substrate.

As can be seen in Figure 3, the Z-shaped Au/Pt hybrid nanorobot was truly tiny, so the manipulation should be accurate and efficient. Figure 4 showed the schematic of manipulation method. According to the strategy of manipulation, except the two manipulators mentioned in Figure 4, the observational instrument in this article was an IX-71 inverted optical microscope (IX71, Olympus, Tokyo, Japan) shown in Figure 5. The manipulation process and the motion of nanorobot were observed by the microscope. The high-sensitivity CCD camera (Neptune100, Watec, Tokyo, Japan) was used to observe the fluorescent image and video on the TV display and record it. The whole experiment platform under the inverted optical microscope is shown in Figure 5.

**Figure 6:**
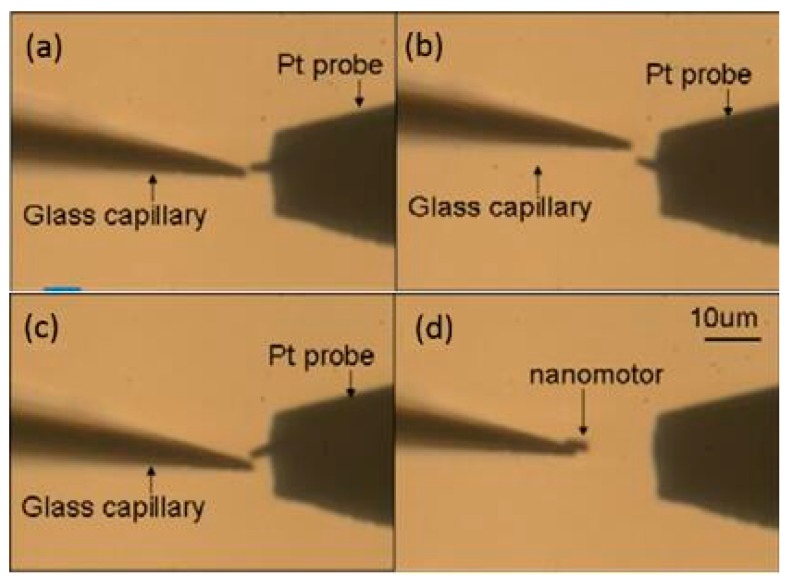
The cut off process of the nanorobot. (**a**) The nanorobot is in the original middle position. (**b**) The nanorobot is pressed upward. (**c**) The nanorobot is pressed downward. (**d**) The nanorobot is cut off from the Pt probe and connects to the glass capillary.

**Figure 7:**
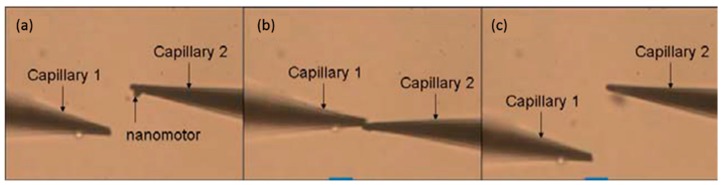
The release process of nanorobot. (**a**) The nanorobot is adhered to capillary 2. (**b**) Pressing capillary 2 with capillary 1 makes it bend. (**c**) Releasing capillary 2 makes it vibrate, and the nanorobot is released from capillary 2. After the releasing of nanorobot, hydrogen peroxide solution was added into the water to obtain the desired concentration.

First, the gold-coated glass capillary was used as an end effector to manipulate the nanorobot. In order to make it easier to observe under the optical microscope, the gold was coated on the surface with a sputtering machine due to the transparent capillary. The glass capillaries both before and after gold sputtering are shown in Figure 5c. The tip was about 1 μm which was small enough to finish the task with high precision. First, the Z-shaped Au/Pt hybrid nanorobot was cut off in water. The critical point was making use of the fatigue failure of platinum, because of the good ductility of platinum. Thus, the cycle stress was applied on the nanorobot by pressing it from both directions: from bottom to top and from top to bottom. The cut-off process was shown in Figure 6. After breaking from the main body, the nanorobot was attached to a glass capillary by the adhesion force which was dominant at the nanometer scale, as shown in Figure 6d. After cutting off the nanorobot from the main body of the Pt probe with the above-mentioned glass capillary connected with manipulator1 (Figure 4a), the Pt probe stage was removed and the second glass capillary controlled by manipulator2 (Figure 4b,c) was brought in. The process was as follows: using one glass capillary pressed onto the second one adhered with nanorobot, the second capillary would bend because of the pressure. We released the pressure rapidly, and the bent glass capillary would vibrate. Due to the vibration, the nanorobot on it was shaken off of it. Moreover, there was no worry that the nanorobot would fly out the observation area; as a matter of fact, that the surface viscosity drag force was dominant over the inertia force in the liquid environment for the micro-nano-sized object. Thus, the vibrated nanorobot would be decelerated quickly and constrained within the observation area. The release process is shown in Figure 7.

### 3.2. Experiment Result Analysis

#### 3.2.1. Analysis of the Nanorobot

**Figure 8:**
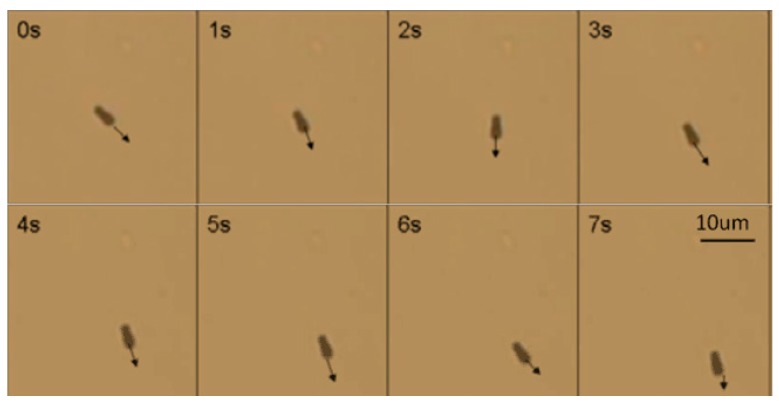
The motion of the nanorobot and the arrow shows the direction.

After the nanorobot was released into the water, the hydrogen peroxide solution was injected into the water. The Au-Pt nanorobot started moving as shown in Figure 8.

Here, the speed of the nanorobot at 14% concentration was analyzed. The corresponding speed was 2.35 μm/s. The calculation of the driving force was based on the result of the experiment and hydrodynamic theory. The dynamic differential equation of motion was based on the model as in Equation (4):

(4) $$m\times a=\sum^{\text{​}}F=F_{drive}-F_{drag},$$

Here, *m* is the mass (kg). *a* is the acceleration (m/s^2^). *F_drive_* is the driving force (N). *F_drag_* is the resistant force (N). The mass of the nanorobot was calculated based on the following equations:

(5) m=ρ×V,

(6) $$V=\pi \times r^{2}\times h,$$

Here, *m* is the mass (g). ρ is the density (g/cm^3^) shown in Table 1. *V* is the volume (cm^3^). *r* is the radius (cm). *h* is the height (cm). The thickness of the Au was 0.1 μm. Thus, the volume of the Au was 0.47 × 10^−11^ cm^3^. The density of the Au was 21.46 g/cm^3^. The volume of the Pt was 2.11 × 10^−12^ cm^3^ and the density of the Pt was 19.3 g/cm^3^. The whole mass of the nanorobot was 5.43 × 10^−11^ g. The acceleration of the nanorobot was calculated based on Equation (7):

(7) $$a=\frac{dv}{dt}\;,$$

The acceleration was 0.335 × 10^−9^ m/s^2^. *F_drive_* is about 3.32 × 10^−14^ N.

#### 3.2.2. Analysis of the Z-Shaped Nanorobot

**Figure 9:**
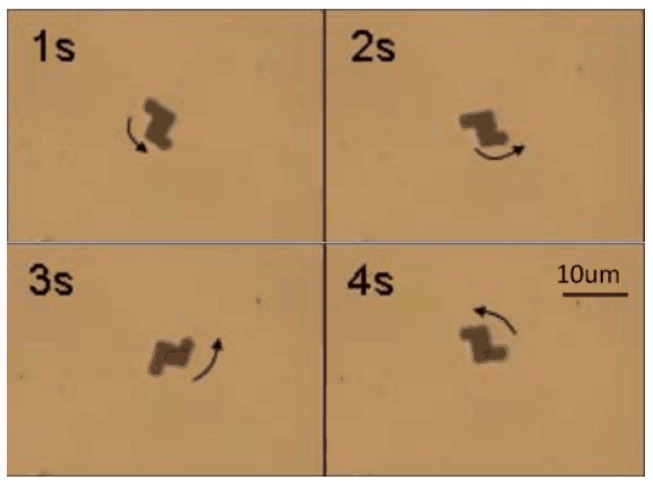
The continuous images of the Z-shaped hybrid Pt/Au hybrid nanorobot.

**Figure 10:**
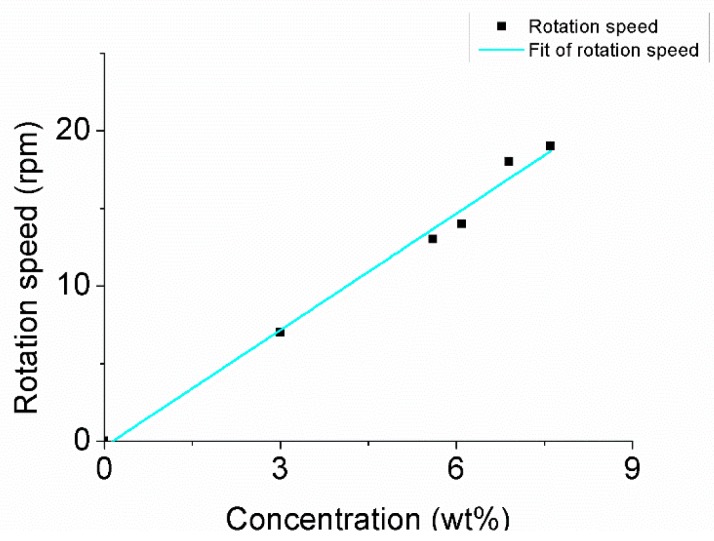
The rotation speed accelerated as the concentration of hydrogen peroxide increased.

After injecting hydrogen peroxide solution into the water, rotational movement was generated, as shown in Figure 9. The nanorobot kept rotating, as Figure 9 demonstrates, in a counter-clockwise direction. The rotational movement was in accordance with the above analysis. Figure 10 indicates the hydrogen peroxide solution concentration influenced the rotational movement of the nanorobot.

The driving force was calculated based on the experimental result and hydrodynamic theory. The dynamic differential equation of motion was based on the model as in Equation (8):

(8) $$J\times \alpha =\sum^{\text{​}}M=(F_{drive}-F_{drag})\times d,$$

Here, *J* is the moment of inertia of the nanorobot (kg·m^2^). α is the angular acceleration (rad/s^2^). *F_drive_* is the driving force (N). *F_drag_* is the resistant force (N). *d* is the arm of force(m). The mass of the nanorobot was calculated firstly. The calculation is based on Equations (9) and (10):

(9) m=ρ×V,

(10) V=A×δ.

Here, *m* is the mass (kg). ρ is the density (kg/m^3^). *V* is the volume (m^3^). *A* is the surface area (m^2^). δ is the thickness (m).

**Figure 11:**
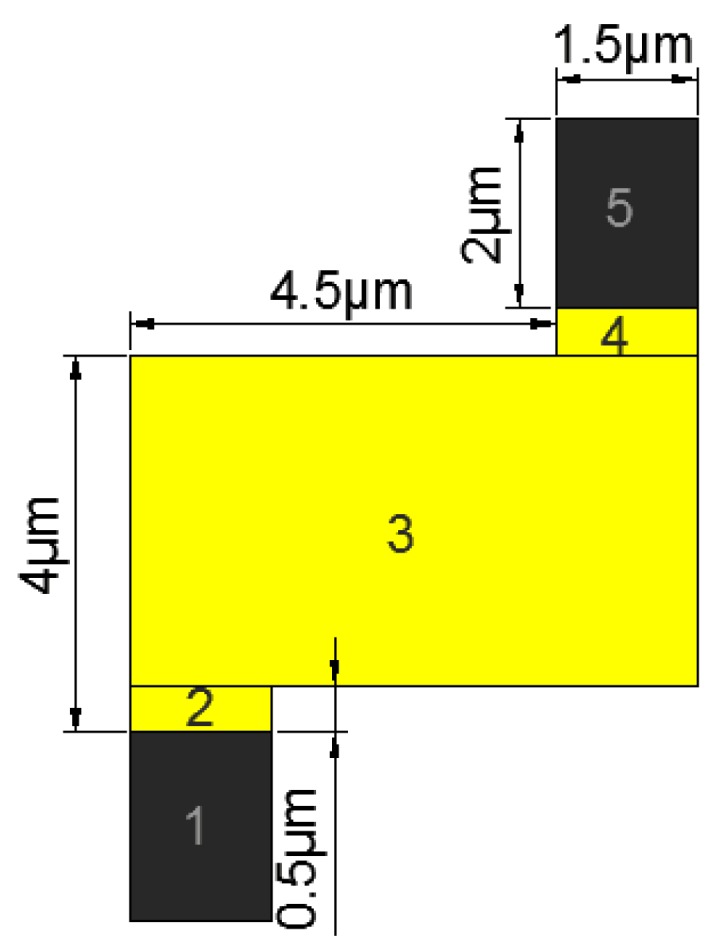
The division of the nanorobot.

The area of the nanorobot was divided into five parts to calculate the moment of inertia of the nanorobot, as shown in Figure 11.

**Figure 12:**
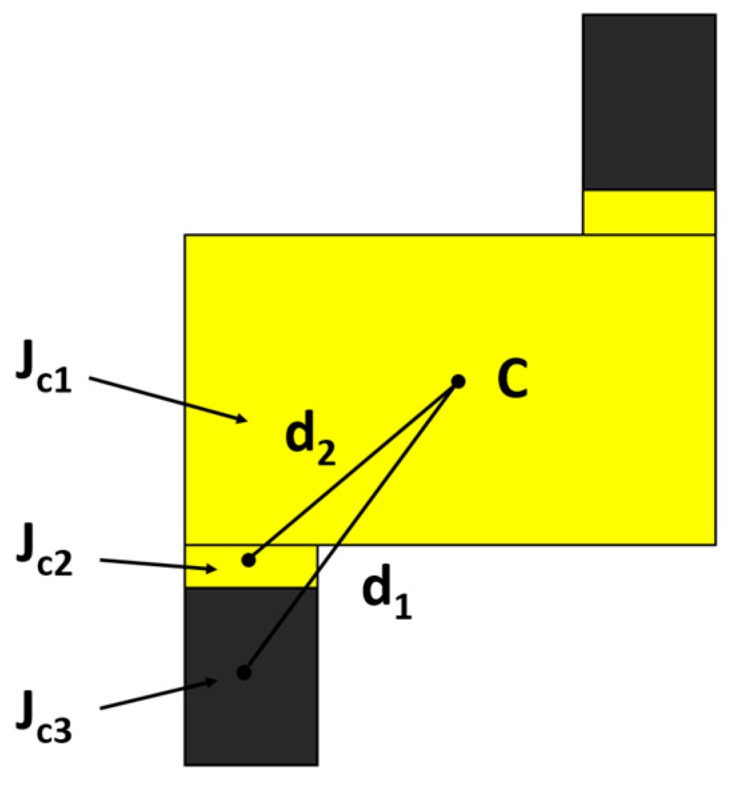
The moment of inertia of each part and the parallel axis theorem.

Due to the origin-symmetric shape, only three parts (1, 2, and 3) needed to be calculated. The mass of the nanorobot was obtained from Equation (9), where ρ is the density of each part and *V* is the volume of each part. The volume was calculated from Equation (10), where A was the surface area of each part and δ was the thickness of each part. The density of the platinum equaled 21.45 g/cm^3^ and the density of the gold was 19.30 g/cm^3^. The dimensions of the nanorobot are shown in Figure 11. The thickness of each part was equal which was 1.5 m. The thickness of the nanorobot was 0.1 m. The mass of each part was obtained: *m*1 = 9.65 × 10^−11^ g, *m*2 = 2.60 × 10^−11^ g, *m*3 = 66.21 × 10^−11^ g. The whole mass was 78.47 × 10^−11^ g. The moment of inertia of each part was obtained from Equations (11) and (12), where *m* is the mass of each part. *a* and *b* are the length and width of each part, respectively. The moment of inertia is calculated by: *J_c_*_1_ = 5.03 × 10^−19^ g/cm^2^, *J_c_*_2_ = 0.54 × 10^−19^ g/cm^2^, and *J_c_*_3_ = 266.23 × 10^−19^ g/cm^2^. Then the parallel axis theorem was used to move all *J_c_* to the center of mass *C*, as shown in Figure 12. The whole moment of inertia was 7.22 × 10^−17^ g/cm^2^.

(11) $$J_{zC}=\frac{m}{12}\times \left(a^{2}+b^{2}\right),$$

(12) $$\;J_{z}=J_{zC}+m\times d^{2}.$$

**Figure 13:**
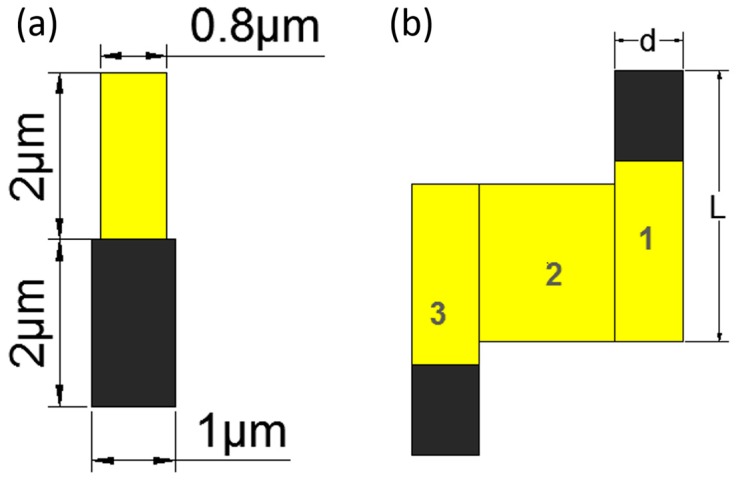
(**a**) The shape of the nanorobot; and (**b**) the division of the Z-shaped nanorobot.

The Z-shaped nanorobot was divided into three parts shown in Figure 13. The driving force was generated by parts 1 and 2. The mechanism of the Z-shaped nanorobot was the same as the nanorobot [11]. The shaped and dimensions of the nanorobot are shown in Figure 13.

The resistant dragging force of the nanorobot was estimated by Equation (13) [21]:

(13) $$F_{drag}=\frac{4\times \pi \times \mu \times L}{\ln\left(\frac{2\times L}{d}\right)+0.5}\times v,$$

*L* and *d* are the length and width of the one part of the nanorobot as shown in Figure 13. μ is the viscosity of the liquid and the viscosity of the hydrogen peroxide was 1.24 × 10^−3^ N/m^2^. *v* is the speed of the nanorobot. *v* is 2.35 μm/s in 14% concentration of hydrogen peroxide. *F_drag_* is obtained from Equation (13). *F_drag_* was 3.32 × 10^−14^ N. The mass of the nanorobot is 5.43 × 10^−11^ g. Thus, the resistant dragging force of the Z-shaped nanorobot was calculated in the same way. *L* is 6 μm and the *d* is 1.5 μm. *v* is 2.25 μm/s at a concentration of 6.9%. After calculating, *F_drag_* is 9.76 × 10^−14^ N. The order of magnitude of the moment of inertia was 10^−17^ g/cm^2^. Since the angular acceleration of the nanorobot was compensated by the order of magnitude, the left of Equation (13) was much smaller than the right. *J* was ignored. Thus. Equation (13) was obtained. Lastly, *F_drive_* (the hydrogen peroxide concentration at 6.9%) was obtained, which is 9.76 × 10^−14^ N.

**Figure 14:**
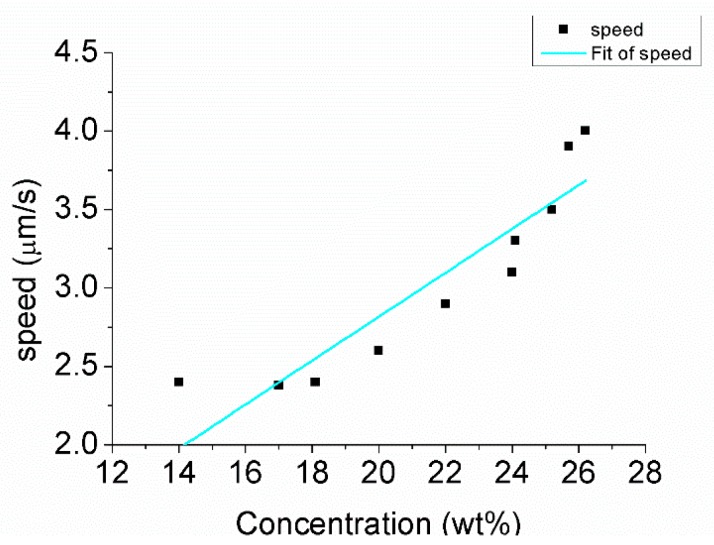
Speed of the nanorobot at different concentrations.

The motion direction of the nanorobot is irregular owing to the Brownian motion [22]. The control experiment, illustrated in Figure 14, was used to demonstrate the influence of the concentration of the hydrogen peroxide solution. Decomposition of the hydrogen peroxide solution is a first-order reaction. The reaction rate is first power proportional to the reactant concentration:

(14) $$r=k\times C_{mol}$$

Here, *r* (mol·L^−1^·s^−1^) is the reaction rate. *k* (s^−1^) is the first-order rate constant which is about 1.01 × 10^−3^/s. *C_mol_* (mol/L) is the concentration of the solution. The wt % was converted into mol/L by Equation (15):

(15) $$C_{mol}=\frac{C_{wt}\times \rho \times 1000}{M}\approx 33\times C_{wt}$$

Here, *C_wt_* (wt %) is the concentration of the solution. ρ (g/mL) is the density of H_2_O_2_, which was 1.13 g/mL. *M* (g/mol) is the molar mass, which is 34 g/mol.

The number of the reacting H_2_O_2_ was obtained by Equation (16):

(16) $$v_{e}=2\times k\times \frac{C_{wt}\times \rho \times 1000}{M}\times Na$$

Here, $v_{e}$ was the velocity of the reacting hydrogen peroxide. *V* (L) is the volume of the hydrogen peroxide solution. *t* (s) is the reaction time. *Na* is Avogadro’s constant, which is 6.022 × 10^23^.

The current density is obtained by Equation (17):

(17) $$J=\frac{I}{A}=\frac{nvqs}{A}=v_{e}Lq$$

Here, *J* is the density of the current. *I* is the current. *A* is the cross-section area of the nanorobot. *v* is the velocity of the electron. *q* is the charge of electron which is 1.6 × 10^−19^. *L* is the length of the nanorobot.

The electron field is obtained by Equation (18) [23]:

(18) $$E=\frac{J}{K}$$

Here, *K* is the bulk solution conductivity which is 1.7 × 10^−8^ Ω^−1^·s^−1^. The relationship between the electric field and velocity is illustrated in [23] as:

(19) $$v=\frac{\delta_{rod}\varepsilon E}{\mu }$$

Here, the velocity (*v* (m/s)) of the nanorobot is related to the electric field (*E*). The zeta potential ($\delta_{rod}$ (B/V)) of the particle is approximately 20 mV [23], with known values for dielectric permittivity and viscosity of the solution (7.08 × 10^−10^ C^2^/(J·m) and 1.0 × 10^3^ N·s/m^2^, respectively). Equation (20) was obtained:

(20) $$v\approx 0.21\times C_{wt}$$

The order of magnitude of the velocity is in accord with the experimental statistics. However, the scale factor 0.21 was larger than 0.14, fitted by experimental statics. 

As shown in Figure 13b, The Z-shaped nanorobot is based on linear nanorobotics. The size of the Z-shaped nanorobot was larger. The scale factor was obtained by half part was 2.71. The rotation speed ω was obtained by Equation (21):

(21) $$\omega \approx 2.71\times C_{wt}$$

Here, ω is the rotation speed. The scale factor 2.71 was larger than 2.5, fitted by experimentation.

A deviation exists between the experimental statistics and theory calculation. The theory calculation of the relationship was in an ideal situation. The experimental situation is not stable due to Brownian motion, decreasing concentration, etc.

## 4. Conclusions

The fabrication and manipulation of the nanorobot was shown in this article. After injecting the hydrogen peroxide solution, the motion was observed by optical microscope. The driving force of the Z-shaped Au/Pt hybrid nanorobot is formed by self-electrophoresis. Two opposite forces on different sides and moment arms formed a couple which drive its rotation. The concentration of the hydrogen peroxide solution changed from 3.0% to 7.6%, and the rotation speed was raised from 7 rpm to 19 rpm. According to the experimental parameters and the experimental data, *F_drive_* was analyzed and (1) the rotational movement is according with the driving mechanism: the moment of inertia is generated by two reverse forces with an arm of force between two sides of the Z-shaped Pt/Au hybrid nanorobot’s two sides; (2) the driving force is related to the concentration of the hydrogen oxide solution; and (3) the order of magnitude of the driving force was 10^−14^ N. The calculation was based on the experimental result, hydrodynamic theory, and the dynamic differential equation of motion.

## Figures and Tables

**Table 1:** Density Table.

Material	Densities (g/cm^3^)
Platinum (Pt)	19.3
Gold (Au)	21.46

## Abbreviations

**Symbols**	**Meanings**
*M*	torque (N·m)
*d*	arm of force or width (m)
*F*	force (N)
*I*	electricity (A)
*m*	mass (g)
*a*	acceleration (m/s^2^)
ρ	density (g/cm^3^)
*V*	volume (cm^3^)
*r*	radius (cm)
*h*	height (cm)
*v*	velocity (m/s)
*t*	time (s)
*J*	moment of inertia (kg·m^2^)
α	angular acceleration (rad/s^2^)
*A*	surface area (m^2^)
δ	thickness (m)
μ	viscosity (N/m^2^)
*L*	length (m)
ω	rotation speed (rpm)
*C*	concentration of solution (wt %)
